# Burnout due to COVID-19 pandemic in frontline healthcare workers from low- and middle-income countries: A systematic review

**DOI:** 10.1192/j.eurpsy.2023.868

**Published:** 2023-07-19

**Authors:** G. Mammadzada, A. Manucheri-Lalen

**Affiliations:** 1 Azerbaijan Psychiatric Association; 2 Department of Psychiatry, Azerbaijan Medical University, Baku, Azerbaijan

## Abstract

**Introduction:**

The coronavirus pandemic was declared one of the deadliest pandemics in the world’s history, surpassing even the 1918 flu pandemic in some countries. The speed with which the virus spread around the world did not leave the slightest chance to prepare even for protected health systems of developed countries, let alone countries with fragile health systems, experiencing a large number of problems at work even at regular times. Although burnout is included in the ICD-11 classification only as a syndrome caused by occupational stress and not a disease, unrecognized and unaddressed, it has the capacity to transform into a wide range of mental disorders, including depressive and anxiety disorders. This poses a threat to healthcare workers and patients, so it is very important to understand the scale of the problem and the factors that can play protective and triggering roles.

**Objectives:**

Our objective was to evaluate point-prevalence of burnout syndrome among healthcare workers from LMIC during the COVID-19 pandemic.

**Methods:**

We conducted a systematic search in Ovid MEDLINE, Embase and PsycInfo, as well as a manual search in PubMed, Google Scholar and Grey Literature to select studies describing burnout prevalence in healthcare workers from low-and middle-income countries during the pandemic. We used a wide range of subject headings as well as main keywords, such as “mental health”, “burnout”, “coronavirus”, “health personnel”, “depression”, “anxiety”, “developing countries” and their synonyms study design search had no limitations and included cohort, case-control, cross-sectional and randomized controlled studies. All identified studies were screened and a final sample of relevant studies was used for data extraction and quality appraisal.

**Results:**

Data was extracted from 15 studies and included 21,007 individuals from eight countries. The proportion of female healthcare workers in our sample was 69% and the overall mean age of participants was 34.3 (2.3). Burnout prevalence varied from 3.62% to 90.1%. Gender, age, workload-related factors and institutional support were mentioned among the most important aspects influencing the burnout level. Clinical heterogeneity was a major drawback of our study as cut-off points for overall burnout and burnout sub-scales were inconsistent even among studies using the same inventory.

**Conclusions:**

It is of critical importance to adopt a wide range of burnout preventive measures for healthcare workers, in particular, easy access to mental health support services, proper organization of human force and more comprehensive educational training in emergency situations. Furthermore, it is suggested, that researchers use a more precise and evidence-based approach to the use of measurement tools in order to increase the reliability of their work and recommendations.

**Disclosure of Interest:**

None Declared

